# Back-calculating the incidence of infection of leprosy in a Bayesian framework

**DOI:** 10.1186/s13071-015-1142-5

**Published:** 2015-10-22

**Authors:** Ronald E. Crump, Graham F. Medley

**Affiliations:** Warwick Infectious Disease Epidemiology Research, School of Life Sciences, Gibbet Hill Campus, The University of Warwick, Coventry, CV4 7AL UK; Public Health & Policy, London School of Hygiene and Tropical Medicine, London, WC1E 7HT UK

**Keywords:** Leprosy, Hansen’s disease, Back-calculation, Forecast, Surveillance, Thailand, Elimination

## Abstract

**Background:**

The number of new leprosy cases reported annually is falling worldwide, but remains relatively high in some populations. Because of the long and variable periods between infection, onset of disease, and diagnosis, the recently detected cases are a reflection of infection many years earlier. Estimation of the numbers of sub-clinical and clinical infections would be useful for management of elimination programmes. Back-calculation is a methodology that could provide estimates of prevalence of undiagnosed infections, future diagnoses and the effectiveness of control.

**Methods:**

A basic back-calculation model to investigate the infection dynamics of leprosy has been developed using Markov Chain Monte Carlo in a Bayesian context. The incidence of infection and the detection delay both vary with calendar time. Public data from Thailand are used to demonstrate the results that are obtained as the incidence of diagnosed cases falls.

**Results:**

The results show that the underlying burden of infection and short-term future predictions of cases can be estimated with a simple model. The downward trend in new leprosy cases in Thailand is expected to continue. In 2015 the predicted total number of undiagnosed sub-clinical and clinical infections is 1,168 (846–1,546) of which 466 (381–563) are expected to be clinical infections.

**Conclusions:**

Bayesian back-calculation has great potential to provide estimates of numbers of individuals in health/infection states that are as yet unobserved. Predictions of future cases provides a quantitative measure of understanding for programme managers and evaluators. We will continue to develop the approach, and suggest that it might be useful for other NTD in which incidence of diagnosis is not an immediate measure of infection.

## Background

Despite a large reduction in prevalence over the last half century, leprosy remains a public health issue in many countries [1]. In 2013 there were 215,000 new cases of leprosy detected and reported worldwide, with 96 % of these being detected in the 18 countries with the highest burden [2]. Leprosy is caused by the bacterium *Mycobacterium leprae* and this infection can be cured through the use of multidrug therapy (MDT). The drug regimen is dependent upon the classification of the case: paucibacillary cases receive six months of treatment with two-drug MDT while multibacillary cases are treated for 12 months with three-drug MDT. Multibacillary cases are identified as those with more than five skin lesions; paucibacillary cases have five skin lesions or fewer. Outside of the Americas there is little published evidence of transmission to humans from non-human reservoirs of infection [3]. Taking this as evidence that non-human reservoirs are not an important source of transmission, then removing clinical, or sub-clinical, infection from people by treatment should reduce the incidence of infection.

In 1991, the World Health Assembly resolved to eliminate leprosy as a public health problem by the year 2000 [4]. The practical definition of this target was a prevalence of less than 1 registered case per 10,000 of the global population. Progress towards the target was helped by the free provision of multidrug therapy (MDT) from 1995 through the World Health Organisation (WHO), initially by the Nippon Foundation and since 2000 by Novartis. The global target was achieved, but leprosy remains an important public health issue at a national level for a number of countries and in some regions of other (previously high burden) countries [5, 6].

Early diagnosis and use of MDT are the principle tools used to combat leprosy [1]. The social stigma associated with leprosy remains a barrier to early diagnosis, and education is seen as an important step to overcome this. The bacillus Calmette-Guérre (BCG) tuberculosis vaccine also has a protective effect against *M. leprae* infection [7], although the relative contribution of these interventions to current control has not been quantified.

The latest WHO target for leprosy is a reduction in the number of new cases with grade 2 disability to below one per million of the global population by 2020 [5]. Grade 2 disability involves deformity of the hands or feet, or severe visual impairment. The national number of new cases with grade 2 disability is one of the variables reported annually to the WHO, along with the total number of new cases and the number of new multibacillary cases.

We consider elimination as the process of reducing the incidence of infection until transmission is zero. The aim of modelling in the context of elimination is two-fold. First, to provide quantitative forecasts of future cases based on data and current understanding. Such forecasts (made public) are essential as a test of programme management and evaluation, and are an early warning of changes in programme effectiveness. Given the long and variable time periods between infection and disease in leprosy, the incidence of disease is a biased measure of the incidence of infection (unless the infection rate is constant). For example, when incidence is exponentially decreasing, there is a bias towards individuals who have longer infection times [8]. Consequently, changes in incidence of disease require interpretation before they can be used to infer changes in incidence of infection. The second, and most common, use of modelling for elimination is the prediction of impacts of changes to control programmes, this type of modelling has been applied to leprosy [9]. Here we concentrate on the first of these aims: forecasting and interpretation. The method we have chosen to use is back-calculation [10], which provides estimates of past incidence of sub-clinical infections, current prevalence of (undiagnosed) sub-clinical and clinical infections and short-term forward predictions of the numbers of new detected and reported cases. Note that even if transmission is halted, cases will still arise in the future as infected individuals progress through disease stages.

Back-calculation [10] has been used to study the infection dynamics of a variety of infectious diseases with long and variable incubation period distributions, i.e. the time between infection and diagnosis, for example HIV/AIDS, BSE, vCJD, and also cancer. Each observed diagnosis is the result of a previous infection. Therefore, given information on diagnoses and the incubation period distribution (IPD), empirical estimates can be made of the number of infections per time interval. The diagnoses made during one interval are a mixture of individuals infected at different times in the past; for example, individuals diagnosed in 2015 might include people infected in the years 1990 to 2010, depending on the infection rates in those years, and the IPD.

For discrete time periods, the basic back-calculation equation is:

$$ {D}_i={\displaystyle \sum_{j=1}^i}{I}_j{f}_{i-j} $$

where *D*_*i*_ is the number of new diagnoses in the *i*^th^ time interval, *I*_*j*_ is the expected number of infections in the *j*^th^ time interval and *f*_*i*−*j*_ is the probability that the time between infection and diagnosis is *i*-*j* time intervals. In the simplest case *f*_*i*−*j*_ comes directly from the IPD. The logic of back-calculation is that given *D* and *f*, the convolution above allows estimation of *I*. Here, we adopt a Bayesian approach to estimate posterior densities, with a concentration on estimation of variability in *I* as much as central locations, and so include some variability in the time period distributions, *f*, and estimate some relative factors. It is impossible to estimate both *I* and *f* simultaneously because of non-identifiability, but we can use prior knowledge and external data to provide estimates of *I* given uncertainty in *f*.

This basic model can be modified to reflect the natural history of the disease under study. For example, AIDS diagnosis was originally modelled as the end-point for disease [10] but this has subsequently been refined to incorporate both AIDS and HIV diagnoses as well as a number of other intermediate disease states based on CD4 count [11].

Previous uses of back-calculation have concentrated on epidemic or endemic situations (i.e. where *I* is increasing or constant). In contrast, leprosy is globally decreasing, and back-calculation might be more generally useful in disease control situations approaching elimination. In the case of leprosy, the time to diagnosis also depends on the public health system in that more active case finding will result in faster diagnosis. Consequently, we will consider time-varying hazards associated with the distribution of time from onset of symptoms to diagnosis, as well as time-varying rates of infection.

## Methods

The endemic nature of leprosy and it’s long incubation period mean that data on individuals’ time of infection are difficult to obtain. Consequently, information on the IPD and the distribution of the time period from onset of clinical symptoms to detection, the detection delay distribution (DDD), must be obtained independently and then incorporated into the back-calculation. Both the fitting of distributions to time periods and the back-calculation were implemented in a Bayesian Markov Chain Monte Carlo (MCMC) setting. The program Just Another Gibbs Sampler (JAGS) [12] was used, with the R statistical environment [13] acting as the front end.

## Fitting time period distributions

### Data on time of infection, onset and diagnosis

Data on 49 individual cases of leprosy with short exposure periods were extracted from the literature [14–20]. These were mostly military service personnel who spent known, limited periods of time in endemic countries before returning to live in non-endemic communities and subsequently being diagnosed.

### Modelling time period distributions

For each individual, the observed endpoints for each time period (onset of symptoms and diagnosis) were modelled as *t*_*o*_ = *t*_*i*_ + *p*_*i*:*o*_ and *t*_*d*_ = *t*_*i*_ + *p*_*i*:*o*_ + *p*_*o*:*d*_, respectively, where *t*_*i*_ was the unobserved time of infection and *p*_*i*:*o*_ and *p*_*o*:*d*_ were the periods from incubation to onset and from onset to diagnosis. Infection time was a uniformly distributed variable between the start and end of exposure and the two time periods were gamma distributed random variables. The observed values, *t*_*o*_ and *t*_*d*_, were both left and right censored, with onset and diagnosis dates taken as being known to within some time interval. The Gibbs Sampler was used to obtain samples from the posterior probability distributions of the shape and rate parameters of the gamma distributions.

## Back-calculation

### National incidence of new leprosy cases

**Fig. 1:**
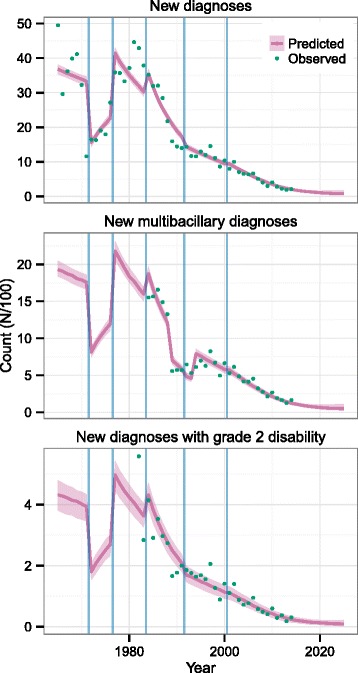
Observed and predicted numbers of new leprosy cases, new multibacillary cases and new cases with grade 2 disability by year for Thailand. *Predicted values are modes and 95 % highest posterior density intervals. The cut-offs between different diagnosis effort/effectiveness levels are indicated by blue vertical lines*

The data used consisted of annual numbers of new leprosy cases, new multibacillary leprosy cases and new leprosy cases with grade 2 disability reported at the national level for Thailand. These data were taken from published reports [2, 21–28]. The variables reported to the WHO have changed over time, resulting in the number of new cases detected, number of new cases with grade 2 disability and the number of new multibacillary cases being available from 1965, 1982 and 1984, respectively. Data on the three variables were available up to 2014. Since 1993, cases of multibacillary leprosy have been identified via case classification (with >5 skin lesions indicating a multibacillary case) rather than a bacterial index score. The transition from the use of the bacterial index to case classification took place from 1989 to 1993. From 1984 to 1988 the number of new lepromatous leprosy cases identified under the Madrid classification system [29] was used as the count of new multibacillary cases [21]. The observed values are plotted against year in Fig. 1.

### Back-calculation model

The progression from sub-clinical to clinical infection and subsequent diagnosis of leprosy was described by a discrete time period model. Calendar years were used as the discrete time step, in keeping with the annual reporting of national summary information to the WHO. The time period of the analysis was set to start 30 years before the first observed record, to allow the system to generate an appropriate pattern of infection to explain the early observations, leprosy being an endemic disease.

The expected incidence of infections in year *b* was simulated with a random walk process. The observed number of infections in year *b* was modelled as:

$$ {I}_b\sim \mathrm{Poisson}\left({\lambda}_b\right) $$

where prior distributions for the λ_*b*_ were

$$ {\lambda}_b\sim \left\{\begin{array}{cc}\hfill \mathrm{Uniform}\left(0,10000\right)\hfill & \hfill \mathrm{if}\ b=1\hfill \\ {}\hfill \mathcal{N}\left({\lambda}_{b-1},{\sigma}^2\right),{\lambda}_b>0\hfill & \hfill \mathrm{if}\ b>1\hfill \end{array}\right. $$

The standard deviation, *σ*, used in the random walk process was set to 30 for the analyses reported in the paper. This was chosen from a number of test runs of the model to provide a smooth downward curve of infections over time while still maintaining some variation. There is no information in the data to allow *σ* to be estimated within the analyses.

Progress from sub-clinical infection to the onset of symptoms was controlled by the IPD, a gamma distribution with constant parameters within a single MCMC chain. The parameters of the IPD were taken from the analyses reported here. From the numbers of sub-clinical infections, the expected number of new clinical infections in any year can be calculated using the IPD:

$$ \mathrm{E}\left[{O}_c\right]={\displaystyle \sum_{b=1}^c}{I}_b{f}_{c-b} $$

Where *f*_*c-b*_ was the probability that time between sub-clinical infection and onset of clinical symptoms was *c*-*b* years. The observed number of clinical infections in year *c* was then *O*_*c*_ ∼ Poisson (E[*O*_*c*_]).

There is no test for sub-clinical leprosy. Therefore, diagnosis occurs subsequent to the onset of symptoms in the model. The probability of being diagnosed in any year given that onset occurred in some known previous year was derived from the onset to diagnosis time period distribution; again using a gamma distribution with constant parameters within a single MCMC chain with the parameters taken from the separate time period fitting reported in this paper. We additionally added a parameter reflecting the effort being put into diagnosis at that time (i.e. scaling the diagnosis hazard for that year).

Variations in the effort put into diagnosis programmes, or the effectiveness of these programmes, is handled by scaling the hazard of being diagnosed in year *d* given that onset of clinical symptoms occurred in year *c* (*h*_*dc*_). The probability of an individual being diagnosed in year *d* given that the onset of clinical symptoms was in year *c* is:

$$ {g}_{dc}={\gamma}_k{h}_{dc}\left(1-{G}_{dc}\right) $$

Where ${G}_{dc}={\displaystyle \sum_{i=1}^{d-1}}{g}_{ic}$, diagnosis effort is modelled by *γ*_*k*_ and *h*_*dc*_ comes from the DDD:

$$ {h}_{dc}=\frac{\mathrm{Prob}\left(c-1\le X<c\right)}{1-\mathrm{Prob}\left(X<c-1\right)} $$

For the Thailand data, six different effort parameters were included to correspond to periods in which the diagnosis effort or effectiveness may have differed [21], as set out in Table 1. The effort parameters, *γ*_*k*_, were modelled using a random walk, such that effort in period *k* was a change from the effort in the previous period. The prior distributions for *γ*_*k*_ were:

$$ {\gamma}_k\sim \left\{\begin{array}{cc}\hfill \mathrm{Uniform}\left(1,{0.25}^2\right)\hfill & \hfill \mathrm{if}\ k=1\hfill \\ {}\hfill \mathcal{N}\left({\gamma}_{k-1},{0.25}^2\right)\hfill & \hfill \mathrm{if}\ k>1\hfill \end{array}\right. $$

**Table 1:** Periods for which the effort or effectiveness of diagnosis may have varied in Thailand, corresponds to parameters fitted

Diagnosis year, *d*	Why
*d* < 1972	In 1971 leprosy treatment moved into general health-care services
1972 ≤ *d* < 1977	On-going training of health-care workers, so possible loss of effectiveness
1977 ≤ *d* < 1984	In 1984 WHO introduced multidrug therapy (MDT), so this period uses general health-care services but no MDT
1984 ≤ *d* < 1992	Both general health-care services and treatment by MDT
1992 ≤ *d* < 2001	1991: global target set of 1 new case per 10,000 of population by 2000
*d* ≥ 2001	Past the cut-off for the global target, possible change in effort

The expected number of diagnoses in year *d* was then:

$$ \mathrm{E}\left[{D}_d\right]={\displaystyle \sum_{j=1}^d}\mathrm{E}\left[{O}_j\right]{g}_{dj} $$

The observed number of new diagnoses in year *i* was a Poisson variable with parameter E[*D*_*i*_], *D*_*i*_ ∼ Poisson(E[*D*_*i*_]). The probability of a multibacillary diagnosis being made in year *d* given that onset of clinical symptoms happened in year *c* was assumed to be unrelated to both the length of time since onset and calendar time; *f*_*dc*_^*M*^ = *ϕ*_*Mi*_*f*_*dc*_, where *ϕ*_*Mi*_ was the proportion of multibacillary cases expected in the *i*^th^ period (*d* < 1989, 1989 ≤ *d* ≤ 1993 and *d* ≥ 1994) and *f*_*dc*_ was the probability of being diagnosed in year *d* given that the onset of clinical symptoms occurred in year *c*. The same prior was used for all three *ϕ*_*Mi*_, *ϕ*_*Mi*_ ∼ Beta(2, 2).

The existence of a relationship between the proportion of grade 2 disability cases and detection delay has been demonstrated previously [30]. This relationship was incorporated into the back-calculation model as a linear regression on detection delay. The probability of a new case with grade 2 disability being made in year *d* given that onset of symptoms happened in year *c* was taken to be *f*_*dc*_^*D*^ = min(*ϕ*_*D*_ + (*d* − *c* + 1)*κ*_*D*_, 1)*f*_*dc*_. Uniform priors were assumed for *ϕ*_*D*_ and *κ*_*D*_, ranging from zero to 0.25 for both parameters.

The uncertainty about the parameters associated with the IPD and DDD was incorporated into the back-calculation via multiple imputation [31] to avoid problems arising from the lack of information in reported case data on the parameters of the IPD and DDD. Multiple MCMC chains were run, with each one using a randomly sampled set of parameters from a multivariate normal approximation to the posterior probability distribution of the IPD and DDD parameters. A total of 100 chains were run, with 50 samples being retained from each. Each chain was run for 250,000 iterations; after 25,000 adaptation and 150,000 burn-in samples one out of every 1,500 samples was retained. The resulting 5,000 samples were merged to provide an overall posterior probability distribution for the parameters of interest. The chains were given equal weight in the merging as they were expected to fit the data equally well.

## Results

### Time period distributions

The IPD and DDD were fitted as gamma distributions with a common rate parameter. In this way the distribution of the sub-clinical infection to detection period is also a gamma distribution with the same rate parameter and a shape parameter equal to the sum of the shape parameters of the two constituent distributions. Table 2 contains a summary of the posterior probability distributions of the three parameters. The parameters are strongly correlated with each other and these relationships were taken into account in the back-calculation. This was done through the use of a multivariate normal approximation to the joint posterior probability distribution of these parameters from which samples were drawn for the multiple imputation runs.

**Table 2:** Summary of posterior probability distributions of the shape parameters for the incubation period and detection delay distributions (*α*_*i*:*o*_ and *α*_*o*:*d*_) and the common rate parameter (*β*)

Parameter	Mode	95 % Highest Posterior Density Interval	Correlation with	
			*α*_*i*:*o*_	*α*_*o*:*d*_
*α*_*i*:*o*_	2.03	1.37 to 2.71		
*α*_*o*:*d*_	1.03	0.73 to 1.37	0.59	
*β*	0.25	0.18 to 0.34	0.80	0.72

The incubation and detection delay periods are assumed to be Gamma distributed with a common rate parameter such that the interval from sub-clinical infection to diagnosis is also Gamma distributed with shape parameter *α*_*i*:*o*_ + *α*_*o*:*d*_ and rate parameter $\beta$

**Fig. 2:**
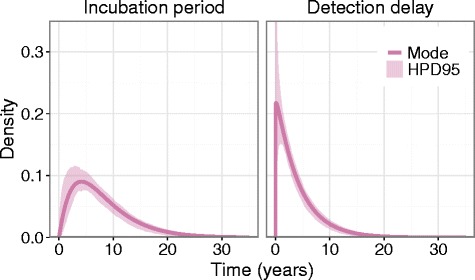
Mode and 95 % Highest Posterior Density intervals (HPD95) of the density of incubation period distribution and detection delay distribution. *The y-axis is truncated. The upper limit of HPD95 for the density of the detection delay distribution is infinite at time zero*

The posterior samples of the time period distribution parameters were used to generate samples of the gamma density for each curve at each time point. These were used to create Fig. 2, which shows the mode and 95 % highest posterior density interval (HPD95) of the two distributions. The modal value of the IPD was 3.8 years (HPD95: 2.10 to 5.64 years). For 95 % of infected individuals, onset occurs by 17.8 years after infection (modal value, HPD95: 15.2 to 22.3 years). Detection occurred for 95 % of clinical infections by 11.4 years post-onset (modal value, HPD95: 9.4 to 14.9 years). The mode of the sub-clinical infection to diagnosis period distribution was at 7.9 years (HPD95: 5.9 to 9.7 years), and 95 % of clinical infections were detected by 23.6 years after sub-clinical infection (modal value, HPD95: 20.4 to 29.2 years).

### Trends in cases

**Fig. 3:**
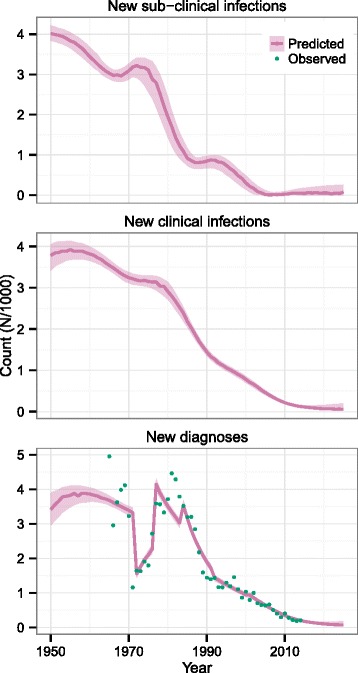
Predicted trends in new sub-clinical infections, clinical infection and detections in Thailand

Figure 3 shows the predicted trends in the numbers of new sub-clinical and clinical infections and reported cases for 1950–2025. The trends in sub-clinical infections and clinical infections are smooth with the clinical infection curve lagging the sub-clinical infection curve slightly.

**Fig. 4:**
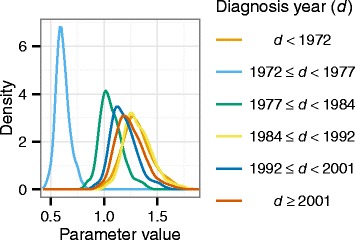
Posterior probability distributions of diagnostic effort/effectiveness parameters in Thailand

The diagnosis hazard in any year is scaled by a diagnostic effort parameter, and this enables the trend in new diagnoses to follow the observed data points reasonably well (Fig. 1). The posterior probability distributions of the six effort parameters are in Fig. 4. During the period where leprosy care was being incorporated into the general health care system and workers were being trained (1972–1977), fewer diagnoses were being made than would be predicted based on the DDD. The modes of the parameter distributions for the other five intervals are above 1, indicating that more diagnoses are made than predicted just using the DDD estimated previously. In particular, the highest diagnostic effort/effectiveness was before 1972, when leprosy care joined the general health care system.

Figure 1 shows the predicted numbers of new cases, new multibacillary cases and new cases with grade 2 disability along with the observed numbers and the cut-offs for different periods of diagnostic effort. The observed values are quite well predicted by the analysis.

The scale which results from the large reduction in the number of new cases over recent decades prevents the variation of the posterior probability distribution being as clear as it would be in close-up, but there is an increase in variation after 2014, when the observations end.

### Prevalence of infection

**Fig. 5:**
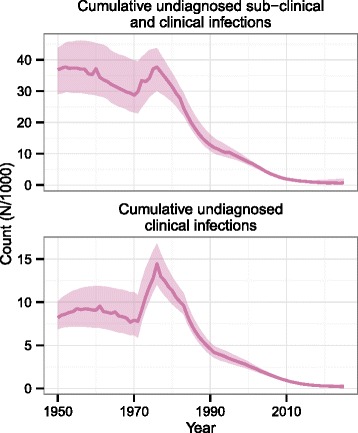
Predicted trends in cumulative numbers of undiagnosed infections in Thailand

In line with the observed decline in new cases, the cumulative burden of undiagnosed infections, both sub-clinical and clinical, has also reduced over time (Fig. 5). As the incidence of diagnoses falls, so the uncertainty in numbers of undiagnosed sub-clinical and clinical infections reduces. The uncertainty in incidence of sub-clinical infection in the very recent past (see Fig. 3) contributes to slightly increasing uncertainty in prevalence in the very recent past. However, the uncertainty in the recent past is largely determined by the choice of *σ* in the random walk.

### Forecasting of diagnoses

**Fig. 6:**
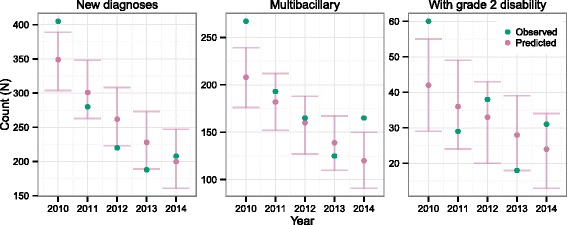
Forward prediction and reported annual numbers of new leprosy cases, multibacillary cases and cases with grade 2 disability from 2010 to 2014. *Posterior probability distributions for predicted values came from running the back-calculation model excluding the observed values for 2010–2014*

The back-calculation analysis was run excluding data from 2010–2014, in order that these could be predicted (Fig. 6). As with the analysis of the full data, 100 samples were taken from a multivariate normal approximation to the posterior probability distribution of the parameters of the IPD and DDD and included via multiple imputation. The predictions of new cases, new multibacillary cases and new cases with grade 2 disability show the correct trend, but a number of observations lie outside of the HPD95 of their predicted values.

## Discussion

We have developed a back-calculation approach to reconstruct the past incidence of infection with leprosy in Thailand. This approach also predicts the numbers of individuals with sub-clinical and clinical leprosy who are yet to be diagnosed, and also future predictions of diagnoses of different types. We believe that these results demonstrate the value of this approach.

### Time period distributions

The use of our estimated time period distributions (IPD and DDD) in the back-calculation analysis is a major source of assumptions. It is assumed that the time period distributions are invariant over time, between sexes and between ages of infected individuals. This is reflected in the transition from sub-clinical infection to clinical infection being completely mediated by the IPD. The assumptions around the DDD are even greater, assuming that detection delays will be far more specific to a country/region and time period. The assumptions about the DDD are mitigated by scaling the hazard associated with diagnosis years to reflect the effort put into, and effectiveness of, case detection. Future work will consider how to extract additional information on time period distributions, particularly as infection rates drop to zero then the last cases are more reflective of the distribution.

### Trends in cases

The annual trends in numbers of new sub-clinical infections and clinical infections were smooth curves, with the new clinical infection trend lagging the sub-clinical infection trend slightly. This is expected from the use of an invariant IPD, with nothing else contributing to the hazard of onset of clinical symptoms in any year. The variation in the number of new infections per year is constrained by the choice of standard deviation associated with the random walk. A number of test runs were performed and a value of 30 was chosen which gave a smooth curve without allowing the new sub-clinical infections to be over-reactive to the observations. It might be more realistic to scale the standard deviation of the random walk in accordance with the number of new infections and this will be investigated in the future. One suggestion is the use of a gamma distribution random walk in which the expected number of new sub-clinical infections in year *b* would be ${\lambda}_b\sim \mathrm{Gamma}\left(k,\frac{k}{\lambda_{b-1}}\right)$. The choice of standard deviation in the current random walk largely controls the amount of uncertainty in the incidence of sub-clinical infection (and hence prevalence of sub-clinical infection) in the very recent past. This is essentially unobservable (i.e. sub-clinical infections yesterday will have no impact on diagnoses for a number of years), so the bounds are effectively 0 to the total population.

The highest estimated diagnostic effort/effectiveness parameter corresponded to the period before leprosy care joined the general health care system, which took place after 1971 [21]. This may reflect a relatively lower level of expertise of diagnostic clinicians in the general health care system leading to longer average detection delays. The diagnostic effort parameters express the effort/effectiveness of the programme relative to a value of one, when the DDD would be the sole determining factor. The effort parameters are therefore dependent upon the specific time period distribution used, which in our case would reflect the predominant health care system at the time of diagnosis of our small sample of individuals with information on the time of sub-clinical infection, onset of clinical symptoms and detection.

The models relating to the occurrence of multibacillary and grade 2 disability cases are very simple, but appear to fit quite well. Additional data would be required to allow investigation of more sophisticated relationships between the incidence of multibacillary or grade 2 disability cases and the detection delay, incubation period and other possible risk factors. The results of analyses of these additional data would then be incorporated into the back-calculation rather than trying to estimate the parameters within the back-calculation from little or no information.

### Prevalence of infection

Note that we are only able to estimate cases of infection that are eventually diagnosed in Thailand. If a significant number of infections resolve spontaneously or do not progress to clinical disease, then our results will underestimate the true numbers of infections. Similarly, if there is significant migration of infections between countries, then our results are less interpretable. In our current model, the cumulative burden of undiagnosed clinical infections is not related to the number or rate of new sub-clinical infections, which might be expected in reality as leprosy is transmissible. This is inherent in the current approach to the analysis; in which infection leads to onset and subsequent diagnosis with no mechanism by which the burden of infection impacts upon the number of future new infections. Future work will consider whether it is feasible to make the incidence of sub-clinical infection a function of the prevalence of clinical, or sub-clinical, infection.

### Forecasting of diagnoses

Our preliminary attempts to forecast cases from 2010–2014 reproduced the decreasing trend but do not fully reflect the variation in the observed values, with several observations falling outside the HPD95 of their predicted values. Given the simplicity of our model, in particular for the prediction of the numbers of new multibacillary cases and new cases with grade 2 disability, this appears to have been quite successful. Future work will include refining the model, including with respect to predicting the numbers of new multibacillary cases and grade 2 disability cases reported. This may require additional data to enable the investigation of parameters associated with some aspects of the model external to the back-calculation, as has been done in other studies [32].

### Reaching the 2020 goal

The goal for 2020 is a reduction in the annual number of new cases with grade 2 disability to below 1 per million of the global population. United Nation predictions of the population of Thailand in 2020 have a 95 % probability interval from 67.8 to 69.4 million, with a median value of 68.6 million [33]. The cut-offs for 1 new case with grade 2 disability per million people are therefore from 67.8 to 69.4. Our observations are that this has already been achieved *within Thailand*, and that the predicted 95 % highest probable density interval of the number of new grade 2 disability cases in 2020 goes from five to 22, with a mode of 13. While it is not possible to extrapolate from predictions for a single country to the global leprosy situation, more meaningful observations about the 2020 goal will be possible when equivalent analyses are run on data from the countries reporting the majority of the world’s new cases for leprosy. This will constitute a major part of the next phase of our research programme.

## Conclusions

Based on this initial analysis the Bayesian back-calculation approach shows much promise as a tool to provide insights into numbers of individuals with sub-clinical or clinical leprosy infections but as yet undiagnosed. Predictions of the expected number of future diagnoses may help to provide some understanding of the elimination process.

Our model remains under development. As well as making improvements around the current analysis, infection incidence by age and gender remain to be incorporated. Differences associated with the infection to onset and onset to diagnosis time period distributions associated with time, age and gender are also being considered.

We suggest that there may be value of using this method of analysis for other neglected tropical diseases in which the incidence of diagnosis is not an immediate measure of infection.

## Abbreviations

DDD: Detection delay distribution
HPD95: 95 % highest posterior density interval
IPD: Incubation period distribution
MCMC: Markov Chain Monte Carlo
WHO: World Health Organisation
